# Forecast of Dengue Cases in 20 Chinese Cities Based on the Deep Learning Method

**DOI:** 10.3390/ijerph17020453

**Published:** 2020-01-10

**Authors:** Jiucheng Xu, Keqiang Xu, Zhichao Li, Fengxia Meng, Taotian Tu, Lei Xu, Qiyong Liu

**Affiliations:** 1 College of Computer and Information Engineering, Henan Normal University, Xinxiang 453007, China; xjc@htu.cn (J.X.); xkqhtu@163.com (K.X.); 2 Engineering Technology Research Center for Computing Intelligence and Data Mining, Xinxiang 453007, China; 3 Engineering Lab of Intelligence Business & Internet of Things, Xinxiang 453007, China; 4 Ministry of Education Key Laboratory for Earth System Modeling, Department of Earth System Science, Tsinghua University, Beijing 100084, China; zhichaoli@mail.tsinghua.edu.cn; 5 Center for Healthy Cities, Institute for China Sustainable Urbanization, Tsinghua University, Beijing 100084, China; 6 State Key Laboratory of Infectious Disease Prevention and Control, National Institute for Communicable Disease Control and Prevention, Chinese Center for Disease Control and Prevention, Beijing 102206, China; mengfengxia@icdc.cn; 7 Institute of Disinfection and Vector Biological Control, Chongqing Center for Disease Control and Prevention, Chongqing 400042, China; taotiantu@sina.cn

**Keywords:** dengue fever, forecast model, long short-term memory, deep learning, transfer learning

## Abstract

Dengue fever (DF) is one of the most rapidly spreading diseases in the world, and accurate forecasts of dengue in a timely manner might help local government implement effective control measures. To obtain the accurate forecasting of DF cases, it is crucial to model the long-term dependency in time series data, which is difficult for a typical machine learning method. This study aimed to develop a timely accurate forecasting model of dengue based on long short-term memory (LSTM) recurrent neural networks while only considering monthly dengue cases and climate factors. The performance of LSTM models was compared with the other previously published models when predicting DF cases one month into the future. Our results showed that the LSTM model reduced the average the root mean squared error (RMSE) of the predictions by 12.99% to 24.91% and reduced the average RMSE of the predictions in the outbreak period by 15.09% to 26.82% as compared with other candidate models. The LSTM model achieved superior performance in predicting dengue cases as compared with other previously published forecasting models. Moreover, transfer learning (TL) can improve the generalization ability of the model in areas with fewer dengue incidences. The findings provide a more precise forecasting dengue model and could be used for other dengue-like infectious diseases.

## 1. Introduction

Dengue is a mosquito-borne tropical disease caused by the dengue virus infection and is a climate-sensitive disease [1,2]. The symptoms include high fever, headache, vomiting, muscle and joint pains, and a skin rash [3]. In a small proportion of incidences, the disease develops into severe cases, resulting in bleeding, low levels of blood platelets, and blood plasma leakage or shock syndrome [1]. The dengue virus (five serotypes of the dengue virus) is transmitted by *Aedes albopictus* and *Aedes aegypti* [4] and is highly sensitive to environmental factors [5,6,7,8]. Meteorological conditions, such as temperature, precipitation, and humidity, have a significant impact on the spread of dengue fever (DF) as the conditions help to increase the population density of *Aedes* [5,6,7,8]. As temperature and precipitation increase, the stages of development of *Aedes* larvae to pupa become shorter, thereby increasing the population growth of *Aedes* [9,10]. Conversely, high temperatures above 35 °C or heavy precipitation may reduce DF transmission by decreasing the survival rate of *Aedes* [9,11]. Beside meteorological variables, socio-economic factors also influence the spread of DF [12].

DF has been a global threat since World War II [13,14]. According to a recent analysis of the global distribution and burden of dengue virus, approximately 390 million dengue cases are reported annually worldwide, especially in Asia and South America [15,16]. The growing number of dengue cases is a huge economic burden [17]. In China, DF reemerged in Guangzhou city in 1978 after the city was free of the disease for more than 40 years [18]. Since then, more regions have reported dengue outbreaks, which include Guangdong, Guangxi, Yunnan, and Zhejiang, and the incidence has increased steadily in recent years [19]. Dengue was one of the most severe public health threats in China, especially when 45,230 dengue cases were reported in Guangdong Province in 2014 [20].

In the absence of effective antiviral agents capable of treating dengue infection, early and accurate forecasts of dengue using different methods might minimize the threat and help the government to implement effective control measures. Numerous studies attempted to predict the incidence of DF using meteorological factors and vector population density or social media data. For example, Shi et al. established a set of statistical models using the least absolute shrinkage and selection operator method to improve the forecasting of dengue in Singapore [21]. Xu et al. used zero-inflated generalized additive models (ZIGAMs) to successfully demonstrate the effects of climatic conditions on the spread of mosquitos and dengue transmission rates [8]. In addition, Li et al. provide accurate dengue prediction by applying the susceptible infected recovered (SIR) epidemic model in mainland China [22]. Chae et al. recognized that some medical organization’s infectious disease reports included delays that can occur in the reporting system and constructed a data-based infectious disease forecast model based on deep learning [23]. However, it is difficult to track DF in a timely manner because of the delayed local mosquito data reports, and most social media data that have received popular attention are not the outputs of instruments designed to produce valid and reliable data amenable for scientific analysis [24].

However, the relationship between dengue cases and meteorological features is highly complex and cannot be easily fitted by the classical time series model. The deep learning method offers more advantages for the health care field as compared with the traditional statistical model [25,26,27,28], and is being actively applied in the prediction the prevalence of infectious disease dynamics [29,30]. Lee et al. showed that artificial neural networks (ANNs) offer a potential benefit in forecasting fluctuations in the mosquito population (especially the extreme values). This method is better than traditional statistical techniques, such as the multiple regression model [31]. Aburas et al. produced encouraging results, with a correlation coefficient of 0.86 for the 2005 period for dengue case prediction in Singapore using ANNs [32]. Truncating the gradient where this does not do harm using memory cells and gate units, LSTM recurrent neural networks can bridge a large number of discrete time steps [33]. Therefore, LSTM is considered as one of the most advanced deep learning architectures for sequence learning tasks, such as speech recognition, or time series prediction [34,35]. LSTM has been used to forecast influenza trends and the epidemics of hand, foot, and mouth disease successfully [36,37]. However, a thorough search of the literature shows that few previous attempts deploying LSTM networks haves been carried out to predict dengue incidence and assess its performance. Current research is more concerned about areas with a high incidence of dengue fever, but it is difficult to perform prediction in areas with fewer dengue incidences. TL is an optimization method used to improve the learning of a new task through the transfer of knowledge from related learning tasks [38]. Correspondingly, TL may be used to improve the learning of models in areas with fewer dengue incidences.

Therefore, this study added lag to the collected data set to consider temporal characteristics. In addition, thorough testing of all the areas selected was performed to examine the model’s robustness. The model prediction performance of this study was verified by comparing it with different prediction algorithms, including a deep learning method and an infectious disease prediction model that uses time series analysis.

Ultimately, by using this study’s obtained results, it should be possible to construct a model that can predict monthly dengue cases in real time. Such a model can not only accurately predict the number of DF cases in areas with a high incidence of dengue fever but also forecast the trend of DF in areas with fewer dengue incidences, which might be helpful for the local government and community to respond early to the disease.

## 2. Study Area Data Collection and Data Preprocessing

### 2.1. Study Areas and Dengue Cases

Case-level records of human dengue incidence data from January 2005 to December 2018 were available in the China National Notifiable Disease Surveillance System (NNDSS). Relevant information for each case was recorded, including basic demographic characteristics (e.g., gender, age, nationality, and residential address) and time of disease-related events (e.g., date of disease onset, diagnosis, and death). All human dengue cases were diagnosed according to the diagnostic criteria for DF (WS216-2008) enacted by the Chinese Ministry of Health [39,40]. Due to the variability of dengue prevalence among the cities, we summarized the data into a monthly scale. Human dengue cases per month (denoted as D) and monthly meteorological data were aggregated for the neural network model training and prediction.

**Figure 1:**
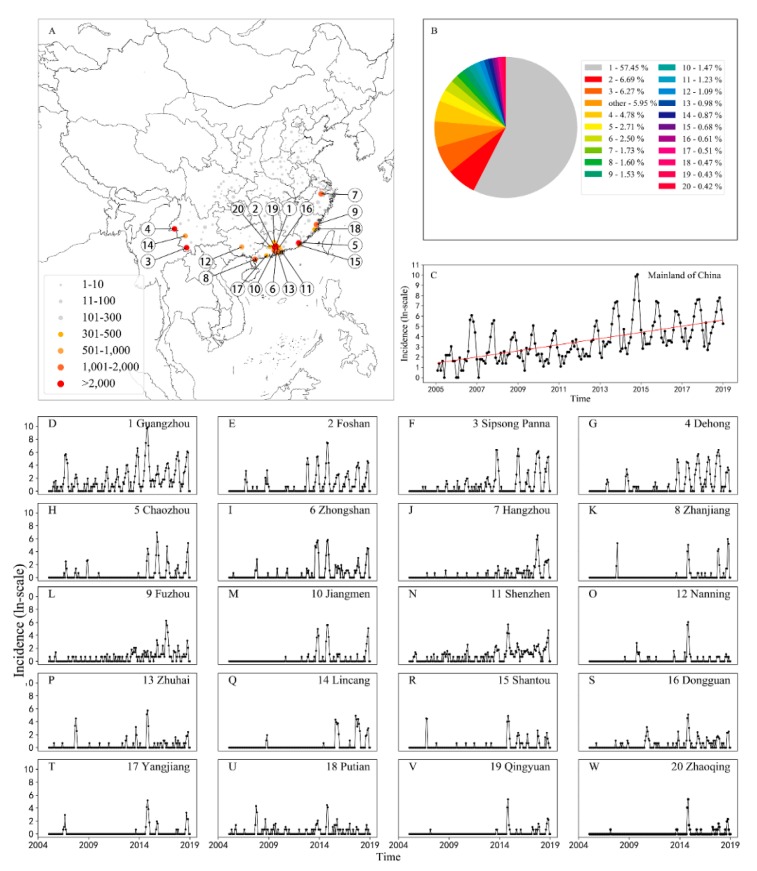
Spatial and temporal distribution of dengue cases in 20 selected cities in mainland China from 2005 to 2018. (**A**) Distribution of dengue cases in China (case numbers are distinguished by color and size according to the magnitude in each city), (**B**) the proportion of cases in each city, (**C**) time series of dengue incidence in mainland China (on the logarithmic scale), and (**D**–**W**) time series of dengue cases in the top 20 cities with the highest dengue incidence (on the logarithmic scale). Based on the Chinese provincial administrative districts public map downloaded from the National Geomatics Center of China (NGCC) website, this figure was produced using the matplotlib basemap toolkit (https://matplotlib.org/basemap/), which is a library for plotting data on maps in Python.

A total of 250 cities in China reported dengue cases from 2005 to 2018 (Figure 1A), and this period shows an increasing trend of dengue human cases (Figure 1C). The top 20 cities (i.e., Guangzhou, Foshan, Sinsong Panna, Dehong, Chaozhou, Zhongshan, Hangzhou, Zhanjiang, Fuzhou, Jiangmen, Shenzhen, Nanning, Zhuhai, Lincang, Shantou, Dongguan, Yangjiang, Putian, Qingyuan, and Zhaoqing) with the highest incidence of dengue were selected as the study areas in this study (Figure 1A). Among these cities, Guangzhou accounts for 57.45% of the total dengue cases in mainland China (Figure 1B). Compared with the dengue epidemic in other cities, the dengue epidemic has obvious periodicity (Figure 1D–W), and the dengue data of Guangzhou were thus chosen to train the pre-training model. Moreover, all of these study areas have frequent economic and cultural communication with the nations of Southeast Asia, where dengue has been hyperendemic for decades [41].

### 2.2. Meteorological Data and Attribute Selection

The meteorological data of these cities were obtained from the National Meteorological Information Center (NMIC). A total of 15 meteorological variables (i.e., extreme wind speed, maximum wind speed, mean wind speed, minimum pressure, maximum pressure, average pressure, mean water pressure, minimum air temperature, maximum air temperature, mean air temperature, average of daily highest temperature, average of daily lower temperature, average of daily precipitation, number of days with rainfall, and average of relative humidity) was retained with no missing values in the raw data. The LSTM model might be overfitted if all variables are used for neural network model training. Overfitting improves the model performances on the training set; however, it works poorly on the test set, indicating that the generalization ability of the model is weak [42]. Thus, attribute selection was used to prevent overfitting and remove redundant attributes.

Attribute reduction for these meteorological variables was carried out using high correlation filtering and low variance filtering. The four variables (i.e., maximum wind speed, extreme wind speed, mean wind speed, and minimum pressure) were deleted as they had the smallest variance in all the study areas. Moreover, the two highest related meteorological variables (i.e., mean minimum temperature, mean air temperature) were removed. In total, 10 valid attributes (i.e., nine meteorological variables and one epidemiological variable) were considered in our study, and the feature parameters are shown in Table 1.

## 3. Method

**Figure 2:**
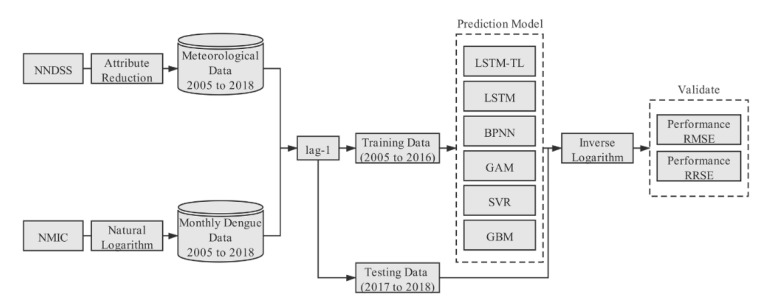
Summarized workflow for the construction of the LSTM-based forecasting model for dengue cases and its comparison with other candidate models. NNDSS: National Notifiable Disease Surveillance System; NMIC: National Meteorological Information Center; BPNN: Back Propagation Neural Network; GAM: Generalized Additive Model; SVR: Support Vector Regression; GBM: Gradient Boosting Machine.

This study constructed an LSTM-based DF prediction model, and compared its performance with other candidate models, i.e., back propagation neural network (BPNN), generalized additive model (GAM), support vector regression (SVR), and gradient boosting machine (GBM). The overall framework of this study is illustrated in Figure 2, and the detailed steps are presented hereafter.

The input data consist of the natural logarithm (NL) of the human dengue cases and meteorological condition variables of the current month. The output data are the log-values of dengue cases in the subsequent month. The normalized data were divided into a training set and testing set. The data from 2005 to 2016 were used as the training set, and the data from 2017 to 2018 were used as a test set, which are the unseen data during the training. The comparison of the model performance proceeded using the predicted value of the inverse logarithm.

### 3.1. LSTM Modeling

**Figure 3:**
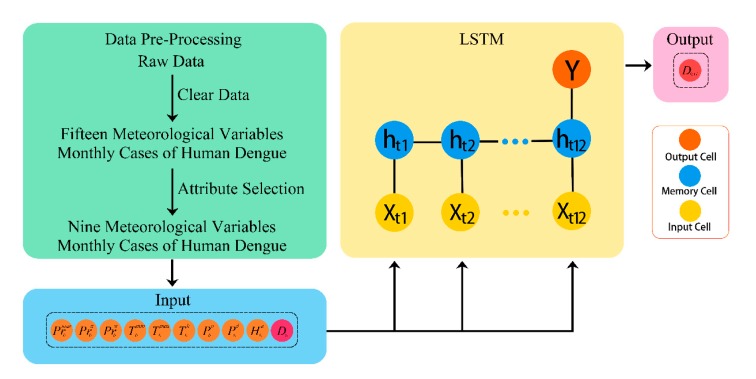
The architecture of the dengue forecast model using the LSTM network.

LSTM, as an advanced intelligent algorithm, enables automatic finding of the characteristics of long-term trends and the short-term fluctuation of time series data. LSTMs belong to the class of improved recurrent neural networks (RNN) and relies on the memory cell with three gating functions incorporated into its construction (Figure S1) [33]. The ability of the LSTM neural network to find the connection automatically between attributes in a time series is derived from learning. A learning process in the neural network model is a weight adjustment from given examples, which makes the network output a true observation without changes in the network structure [32]. The LSTM model consisted of 10 input parameters, such as the monthly mean maximum temperature, monthly average relative humidity, monthly raining days, and the observation taken last month. The $X_{ti}=\left({Pr}_{ti}^{max},{Pr}_{ti}^{a},{Pr}_{ti}^{w},T_{ti}^{min},T_{ti}^{max},T_{ti}^{h},P_{ti}^{a},P_{ti}^{d},H_{ti}^{a},D_{ti}\right)$ is a set of input vector sequence in the same month. The $c=\left(c_{1},c_{2},\ldots ,c_{64}\right)$ is the number of hidden layers in a memory cell. The input time series $X=\left(X_{t1},X_{t2},\ldots ,X_{t12}\right)$ is transmitted to the hidden layers, which contain n memory cells in each one, through weighted connections to compute output Y, the logarithmic of dengue cases in the subsequent month. The inputs are shown in $X_{t1}$ to $X_{t12}$ and the output is shown in Y (Figure 3).

Most of the experiments were performed in Python (version 3.6.8) and run in the hardware environment with 64-bit Windows, a 3.0 GHz, Intel Core i5-8500 CPU. The LSTM network models used in this study were modeled through Tensor Flow (version 1.13.1), which is Google’s released application programming interface for deep learning [43,44].

The architecture has three layers: A single RNN layer with LSTM (hidden layer), which includes 64 memory cells; an input layer; and an output layer (Figure 3). The initial learning rate was left at the value of $1e^{-4}$. Since the transmission cycle of the dengue virus is one year in China, the time step of the LSTM network was set to 12. The root mean squared error (RMSE) in the validation set was smallest when the model input 12 sets of parameters to produce an output of the number of dengue cases in the next month (Table 2). Other Tensorflow defaults included weights’ and biases’ initialization and the activation function for the recurrent nodes. In addition, the regulation was used so that a dropout layer was added between the LSTM and the output layer and the dropout rate was set to 40% to combat network overfitting. The internal weight parameters of the LSTM neural network were adjusted through the adaptive momentum (Adam) optimizer [45]. The training time was 8000 epochs. Our source code is available at https://github.com/KeqiangXu/Dengue_Forecast_Based_on_LSTM.

### 3.2. Candidate Models

For LSTM, we designed two model training routes: One was used to train the LSTM model using the local data only; the second was used to train a pre-train model using data from Guangzhou and train models of other cities using transfer learning (TL). Moreover, the Guangzhou data were selected to train the pre-training model as they contained a large number of DF cases. This was the LSTM model that learns the concepts of mapping the input (meteorological data and dengue cases data) and output data (dengue cases in the next month). The model fit of the Guangzhou data was the starting point for the model of the second city.

In addition, we also computed other models that have been applied in dengue prediction. The BPNN model has shown excellent performance in multivariate time series prediction. For the BPNN model, an optimal parameter (include the number of neurons in the hidden layer and learning rate) was selected to avoid overfitting and improve the predictive performance [32]. For the SVR model, we considered using an ε-SVR approach, which uses a linear kernel function to track dengue dynamics [46]. For the GAM model [47] and the GBM model [48], the parameters of training used the default values in the python package.

### 3.3. Model Validation

Based on the inverse logarithm of model outputs, the model performance and prediction accuracy were measured by RMSE. RMSE is widely used to evaluate continuous variables by measuring the differences between predicted and observed values [49]:

(1) $$RMSE=\sqrt{\frac{1}{N}\sum_{i=0}^{N}{\left(Y_{t}-\bar{Y_{t}}\right)}^{2}},$$

where $Y_{t}$ is the dengue cases of observation for time t, and $\bar{Y_{t}}$ is the number of cases predicted by the model. A smaller RMSE value indicates a smaller difference between the predicted and observed values and indicates a higher prediction performance of the model.

The root relative squared error (RRSE) can be used to evaluate the goodness-of-fit between predicted and observed values. Mathematically, the RRSE is evaluated by Equation (2):

(2) $$RRSE=\sqrt{\frac{\sum_{i=0}^{N}{\left(Y_{t}-\bar{Y_{t}}\right)}^{2}}{\sum_{i=0}^{N}{\left(Y_{t}-U\right)}^{2}}},$$

where U is the average of the observation. The RRSE index ranges from 0 to infinity, with 0 corresponding to the ideal.

In order to fully evaluate the predictive performance of the model, we designed two scenarios. First, we evaluated the prediction accuracy over the last 24 months of each model and compared its performance; second, data from July to November, which covers the peak in dengue incidence in 2017 and 2018, were selected to assess the prediction performance of the model.

## 4. Results

### Comparison of LSTM LSTM-TL and Candidate Models

The statistics of the study show a trend of an increasing number of dengue cases year by year (Figure 1C). Most of the dengue cases occurred in the Pearl River Delta region of Guangzhou, Foshan, Zhongshan, Jiangmen, Shenzhen, and Zhuhai (Figure 1B). In particular, the dengue cases in Guangzhou accounted for more than half of the total dengue cases in China between 2005 and 2018 (Figure 1B).

The LSTM model, by TL training, has a lower RMSE in most cities than the LSTM model by training using only local data. Among the cities, Qingyuan, Dongguan, Shenzhen, Foshan, and Zhongshan are in the same province as Guangzhou, and the distance from Guangzhou is not more than 100 km. The predicted reductions in RMSE in these cities are significant, at 34.6%, 47.4%, 30.3%, 26.9%, and 32.5%, respectively. The RMSE prediction results significantly declined in these cities in the vicinity of Guangzhou because TL can improve the model to some degree, but it is required that the source and target tasks should be the same [38].

**Figure 4:**
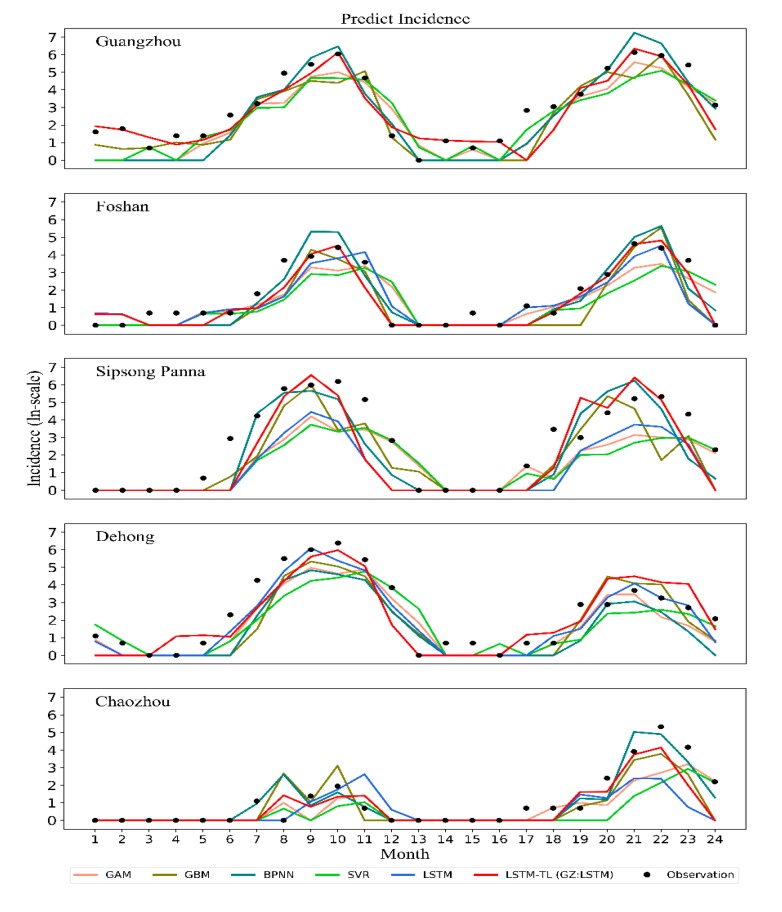
Prediction dengue cases in the last 24 months by the long short-term memory (LSTM) model, back propagation neural network (BPNN) model, gradient boosting machine (GBM) model, generalized additive (GAM) model, and support vector regression (SVR) model. Comparison of 24-month predictions for 2017 to 2018 in Guangzhou, Foshan, Sipsong Panna, Dehong, and Chaozhou which pose a high degree of dengue infection.

The predictive accuracy of dengue cases for each model from 2017 to 2018 and during the outbreak are shown in Table 3. According to the predictive accuracy for the two prediction periods, the LSTM model by TL training has a lower RMSE in most cities than the BPNN model, GAM model, SVR model, and GBM models. Our LSTM method reduced the average RMSE predictions by 12.99% to 24.91% as compared with the estimated dengue cases of other previously published models, and the average RMSE predictions in the outbreak period decreased by 15.09% to 26.82% (Table 3). Notably, the LSTM method reduced the RMSE predictions by 44.48% to 75.56% in Guangzhou (Table 3), which has the highest incidence of dengue fever in China, and the RMSE predictions in the outbreak period decreased by 44.75% to 75.7%. The goodness-of-fit assessment for each model is shown in Table 4. The predicted trend of dengue incidence in the top five cities with high dengue incidence by the LSTM model, GBM model, GAM model, and SVR model from 2017 to 2018 is shown in Figure 4. The predicted trend of dengue incidence in the other 15 cities with high dengue incidence by the LSTM model, GBM model, GAM model, and SVR model from 2017 to 2018 is shown in Figure S2–S4.

## 5. Discussion

This study reviewed the meteorological factors related to dengue occurrence and proposed an LSTM-based model to efficiently predict dengue cases in 20 cities in mainland China. According to our best knowledge, this is the first time that dengue forecasting models were established based on the LSTM network and assessed in mainland China.

Judging from the national legal infectious disease report in the past five years, the incidence of dengue fever in China has continuously been high. According to the predicted data of the model, the government can track dengue dynamics to carry out targeted prevention and control measures. To date, different dengue forecast models have been developed [20,24,50,51]. The Chinese Center for Disease Control and Prevention (CCDC) has introduced the China Infectious Disease Auto-mated-alert and Response System (CIDARS) for the detection of dengue outbreaks, but reports of the spread of dengue by the system are delayed, because this method is dependent on the numbers of notified dengue cases [51]. Some scholars have developed dengue forecast models using climate data, mosquito density data, and dengue case data [20,24]. These models are unable to track dengue fever in a timely manner, and there is room to improve the model’s predictive performance, due to the fact that the local mosquito data cannot be updated quickly in China. However, dengue case data and meteorological data can be updated promptly in relevant departments. Thus, mosquito data were discarded, and monthly dengue cases and meteorological data were chosen to develop a DF prediction model.

Dengue outbreaks in mainland China are often caused by the virus being carried by returning travelers or visitors to China from dengue-endemic areas elsewhere, and most of the dengue cases occur in autumn [52]. In addition, we observed a wide band of numbers of monthly dengue cases ranging from 0 to 18,569 cases, which makes it difficult to predict dengue cases. To obtain accurate forecasting of non-linear time series, such as the prediction of infectious disease, it is crucial to model the long-term dependency in time series data. The periodic patterns spanning multiple time steps are difficult for a typical machine learning method to identify, but this can be achieved by the LSTM network [53,54]. The results in Table 2 showed that the setting of the time step affected the model performance and the model obtained got the best parameters when the time step was set to 12, which suggested that the underlying mechanism of dengue outbreak may be related to long-term climate change. Thus, the LSTM neural network was chosen to develop a DF prediction model that can accurately predict the prevalence of dengue in a timely manner.

In this study, the dataset was relatively small for deep learning models, and the neural network model was less effective for low-resource training, although the climate has been proven as a driving force for DF [8,13], which has a positive effect on the deep learning model, assisting in capturing the law of viral transmission and in predicting the number of cases. However, TL can improve the model to some degree, but it is required that the source and target tasks should be the same [38]. The TL is an optimization method to improve the learning of a new task through the transfer of knowledge from related learning tasks. In our study, TL is applicable in similar climate regions, and it can improve the learning of a new model in areas with fewer dengue incidences through transfer from the already trained model in areas with high dengue incidences.

The predictive accuracy and goodness-of-fit of our LSTM model is superior to the other models in Guangdong, which is China’s most dengue-hit area (Table 3 and Table 4). Further, we compared the neural network models in more cities of China with other previously published models in Table 3. In terms of prediction accuracy, the LSTM neural network model has a lower RMSE in most cities than other models. By the LSTM model being applied in more cities in mainland China, we found that the LSTM failed to capture the characteristics of viral transmission in areas with a low dengue incidence. This problem can be solved using TL in areas in the vicinity of Guangzhou with high RMSE (by LSTM). It can be seen that the LSTM model or the LSTM model trained by transfer learning in the outbreak period was closer to the observations (Table 3).

There have been a large number of relevant documents on the study of the impact of meteorological factors on dengue fever. However, most of the research models are limited to short-term analysis, and the cumulative effect and hysteresis of relevant factors are not considered, which limits this research results. At the same time, the geographical differences and research materials of different spatial scales will bring about a lack of comparability in the research results. Based on the LSTM method, this study achieved an accurate prediction of DF cases for high-risk areas in mainland China, using long-term time series dengue cases and the data of meteorological variables. This method might be used for the large-scale prediction of other dengue-like diseases.

However, our research has some limitations. First, the LSTM model takes a large amount of time for training compared to other machine learning models; however, the impact is not significant since the data collected in this study were from a small-sized dataset. Second, we could not obtain accurate predictions in some cities by using any model in this study, probably because we failed to consider other relevant potential socio-economic factors [55].

## 6. Conclusions

This study proposed an LSTM-based model, which enabled us to efficiently predict monthly dengue cases using meteorological data and dengue cases in 20 cities of mainland China. Several candidate models were also implemented in order to appraise the performance of the LSTM-based model. Briefly, the LSTM-based model could identify periodic patterns spanning multiple time steps in non-linear time series. Moreover, integrating LSTM and transfer learning could improve the prediction accuracy. We conjecture that the proposed LSTM and LSTM-TL model might be used for the large-scale prediction of other dengue-like diseases.

## Figures and Tables

**Table 1:** The feature parameters used in the long short-term memory (LSTM) modeling in this study.

Parameters	Symbol	Unit
Maximum pressure	${Pr}^{max}$	hPa
Average pressure	${Pr}^{a}$	hPa
Average of water pressure	${Pr}^{w}$	hPa
Minimum air temperature	$T^{min}$	°C
Maximum air temperature	$T^{max}$	°C
Average of daily highest temperature	$T^{h}$	°C
Average of daily precipitation	$P^{a}$	mm
Number of days with rainfall	$P^{d}$	D
Average of relative humidity	$H^{a}$	%RH
Human dengue cases per month	D	Ln(case + 1)

**Table 2:** The root mean square error (RMSE) validation set for a different number of time steps in the LSTM model.

Time Step (*i*)	RMSE
i = 1	54.06
i = 2	70.56
i = 3	49.02
i = 4	46.72
i = 6	66.75
i = 12	43.10

**Table 3:** Comparison of model performances using the root mean square error (RMSE). The number before the symbol “/” is the RMSE of the model prediction for the last 24 months, and the number after the symbol “/” is the RMSE of the model prediction in the outbreak period (July to November in 2017 and 2018). TL: Transfer Learning.

City	LSTM-TL	LSTMs	BPNN	GAM	SVR	GBM
Guangzhou		53.29 */82.04 *	217.97/337.62	95.99/148.50	119.46/184.86	113.36/175.28
Foshan	13.36 * /12.19 *	18.28/18.09	58.94/42.98	24.50/34.53	27.51/36.83	39.51/30.02
Sipsong Panna	133.90/207.08	139.54/215.80	112.89/174.46	145.37/224.93	148.70/230.10	118.71 */183.54 *
Dehong	66.86 */102.71 *	85.44/132.00	126.60/195.78	122.05/188.92	135.48/209.82	109.38/169.05
Chaozhou	44.37 */48.57 *	60.01/65.80	56.16/97.19	60.55/104.84	61.63/106.71	55.62/96.25
Zhongshan	7.89*/44.09 *	11.69/44.86	27.95/58.22	22.36/60.15	23.84/62.74	19.41/58.81
Hangzhou	160.71/248.93	160.43/248.51	160.33 */248.35 *	160.39/248.43	160.72/248.94	160.71/248.93
Zhanjiang	80.81 */125.18 *	83.81/129.83	96.38/149.28	89.67/138.91	90.19/139.72	84.57/131.01
Fuzhou	5.01 */7.41 *	5.67/8.44	7.72/11.80	5.05/7.60	5.13/7.70	10.52/16.01
Jiangmen	24.45/37.88	28.51/44.16	23.26 */36.02 *	31.19/48.26	32.37/50.11	31.70/49.11
Shenzheng	14.34 */21.69 *	20.58/31.59	20.28/31.18	23.87/36.74	24.30/37.43	24.58/37.77
Nanning	0.94/1.42	1.92/2.13	0.53 */0.75 *	0.58/0.83	0.70/1.03	0.76/1.13
Zhuhai	2.79/4.27	3.27/5.03	3.05/4.68	2.46 */3.76 *	2.62/3.99	10.72/16.60
Lincang	**35.51** */54.88	36.74/56.81	36.40/56.32	36.54/56.56	35.62/55.14	35.82/54.53 *
Shantou	3.62/5.45	3.56/5.36	5.50/8.39	3.54/5.34	3.50 */5.21 *	14.8222.90
Dongguan	2.44 */3.40 *	3.64/5.33	3.03/4.37	2.84/4.18	3.30/4.95	2.80/4.14
Yangjiang	5.87/9.09	6.33/9.80	5.84/8.92	5.63/8.60 *	5.77/8.84	5.62 */8.71
Putian	1.33/1.59	1.80/2.46	1.10 */1.06 *	1.17/1.22	1.13/1.17	3.01/4.48
Qingyuan	1.93 */2.90 *	2.95/4.51	5.92/9.14	2.64/4.03	2.95/4.52	2.28/3.47
Zhaoqing	**2.29** */3.46	2.50/3.80	2.36/3.58	2.58/3.25 *	2.54/3.86	2.82/3.01
Average of RMSE	32.02 */49.59 *	36.50/55.82	48.61/76.28	41.95/66.48	44.37/70.18	42.33/65.74

* and bold indicate the values of the RMSE of this model were the smallest.

**Table 4:** Comparison of the models’ goodness-of-fit using the root relative squared error (RRSE). The number before the symbol “/” is the RRSE of the model prediction for the last 24 months, and the number after the symbol “/” is the RRSE of the model prediction in the outbreak period (July to November in 2017 and 2018).

City	LSTM-TL	LSTMs	BPNN	GAM	SVR	GBM
Guangzhou		**0.3664 ^§^/0.6805 ^§^**	0.5036/0.9843	0.5130/0.9963	0.6296/1.2305	0.6018/1.1709
Foshan	**0.4179 ^§^/0.7060 ^§^**	0.5625/0.9504	0.9096/1.5374	0.6970/1.1666	0.8219/1.3674	0.7043/1.1903
Sipsong Panna	0.7020/1.3122	0.9323/1.7644	**0.6410 ^§^/1.1895 ^§^**	0.9870/1.8794	1.0358/1.9745	0.7965/1.5024
Dehong	0.4651/0.6041	**0.3934 ^§^/0.5244 ^§^**	0.6499/0.8833	0.6003/0.8247	0.7390/1.0209	0.5747/0.7757
Chaozhou	0.6045/0.6741	0.9701/1.0877	**0.4962 ^§^/0.5540 ^§^**	0.8449/0.9507	0.9426/1.0606	0.6816/0.7616
Zhongshan	**0.3102 ^§^/0.3895 ^§^**	0.3740/0.4695	0.5611/0.6579	0.7904/0.9834	0.8673/1.0827	0.6656/0.8342
Hangzhou	1.1071/1.2778	**1.0999 ^§^/1.2696 ^§^**	1.1018/1.2719	1.1037/1.2738	1.1074/1.2782	1.1071/1.2778
Zhanjiang	**0.8545 ^§^/1.0059 ^§^**	0.9014/1.0610	0.9925/1.1683	1.0438/1.2287	1.0590/1.2466	0.8987/1.0579
Fuzhou	**1.0414 ^§^/1.1392 ^§^**	1.1402/1.2520	1.4755/1.6458	1.0569/1.1653	1.0718/1.1775	1.9776/2.1833
Jiangmen	0.5351/0.5995	0.7234/0.8104	**0.5315 ^§^/0.5956 ^§^**	0.8354/0.9305	0.9096/1.0159	0.7484/0.8386
Shenzheng	**0.4997 ^§^/0.5404 ^§^**	0.7227/0.8405	0.7016/0.8248	0.9279/1.0996	0.9593/1.1413	0.9712/1.1462
Nanning	2.9945/3.7608	6.8850/6.3471	1.0997/1.3467	**1.0538 ^§^/1.2870 ^§^**	1.1599/1.4247	1.8182/2.2684
Zhuhai	0.9768/1.1862	1.1120/1.3507	1.0313/1.2526	**0.8988 ^§^/1.0898 ^§^**	0.9257/1.1172	2.9623/3.6012
Lincang	**0.9857 ^§^/1.3449 ^§^**	1.0842/1.4823	1.0668/1.4600	1.0821/1.4838	1.0267/1.4094	1.0573/1.4442
Shantou	1.1179/1.2945	1.1099/1.2849	1.6159/1.8820	1.0772/1.2453	**1.0546 ^§^/1.2160 ^§^**	3.0543/3.5878
Dongguan	**0.6936 ^§^/0.9089 ^§^**	1.1209/1.5226	0.9238/1.2367	0.8586/1.1739	1.0361/1.4236	0.8467/1.1544
Yangjiang	0.9814/1.1232	1.0560/1.2083	0.9630/1.0875	**0.9500 ^§^/1.0731 ^§^**	0.9554/1.0827	0.9572/1.0954
Putian	1.4112/2.0723	2.1944/3.7520	**1.0619 ^§^/1.1335 ^§^**	1.1314/1.3462	1.0688/1.1627	3.7607/6.8112
Qingyuan	**0.7566 ^§^/0.8874 ^§^**	1.1102/1.3084	1.9780/2.3374	1.0723/1.2633	1.1111/1.3094	0.9177/1.0794
Zhaoqing	1.0479/1.2060	1.1026/1.2698	1.1113/1.2799	0.9969/**1.1464 ^§^**	1.1091/1.2773	**0.9070 ^§^**/1.0404
Average of RMSE	**0.9087 ^§^/1.1600 ^§^**	1.2322/1.5210	0.9708/1.2165	0.9261/1.1804	1.3005/1.7411	0.9795/1.2510

^§^ and bold indicate the values of the RRSE of this model were the smallest.

## Supplementary Materials

The following are available online at https://www.mdpi.com/1660-4601/17/2/453/s1, Figure S1: The memory cell structure of the long short-term memory network (LSTM) hidden layer, Figure S2: Prediction dengue cases in the last 24 months by the LSTM model, BPNN model, GBM model, GAM model, SVR model. Comparison of 24-month predictions for 2017 to 2018 in Zhongshan, Hangzhou, Zhanjiang, Fuzhou, and Jiangmen, Figure S3: Prediction dengue cases in the last 24 months by the LSTM model, BPNN model, GBM model, GAM model, SVR model. Comparison of 24-month predictions for 2017 to 2018 in Shenzhen, Nanning, Zhuhai, Lincang, and Shantou, Figure S4: Prediction dengue cases in the last 24 months by the LSTM model, BPNN model, GBM model, GAM model, SVR model. Comparison of 24-month predictions for 2017 to 2018 in Dongguan, Yangjiang, Putian, Qingyuan, and Zhaoqing.
