# Supplementary material for: Forecast of Dengue Cases in 20 Chinese Cities Based on the Deep Learning Method
Source: Int J Environ Res Public Health. 2020 Jan 10;17(2):453. doi: 10.3390/ijerph17020453 (PMC7014037; doi:10.3390/ijerph17020453)
Supplement: Supplementary file 1 [file ijerph-17-00453-s001.pdf]

International Journal of
Environmental Research and Public Health

Supplementary Materials

Forecast of Dengue Cases in 20 Chinese Cities Based on the Deep Learning Method

Jiucheng Xu [1,2,3], Keqiang Xu [1,3], Zhichao Li [4,5], Fengxia Meng [6], Taotian Tu [7], Lei Xu [4,5,6,*] and Qiyong Liu [6,*]

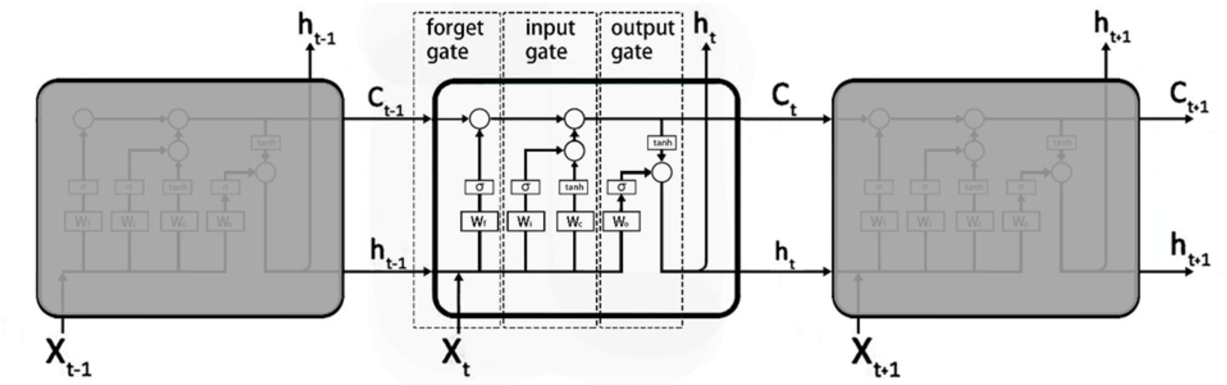

Figure 1. The memory cell structure of the long short-term memory network (LSTM) hidden layer.

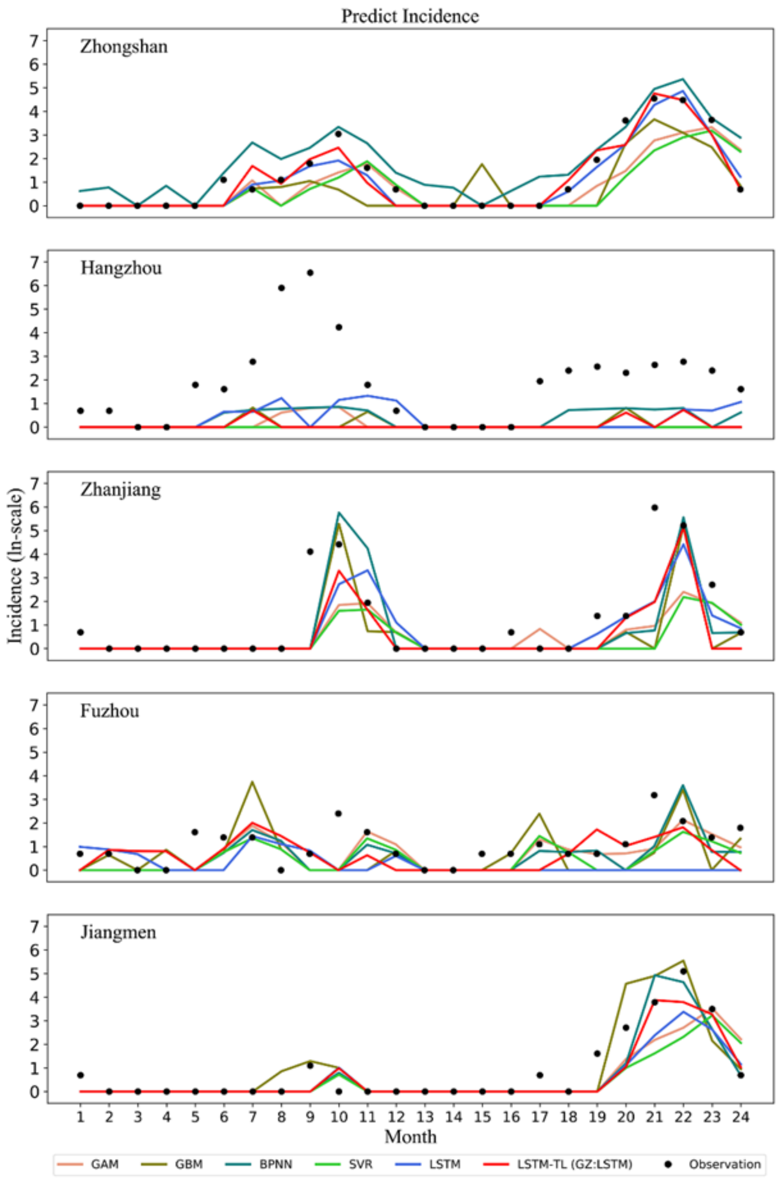

Figure 2. Prediction dengue cases in the last 24 months by the LSTM model, BPNN model, GBM model, GAM model, SVR model. Comparison of 24-month predictions for 2017 to 2018 in Zhongshan,.

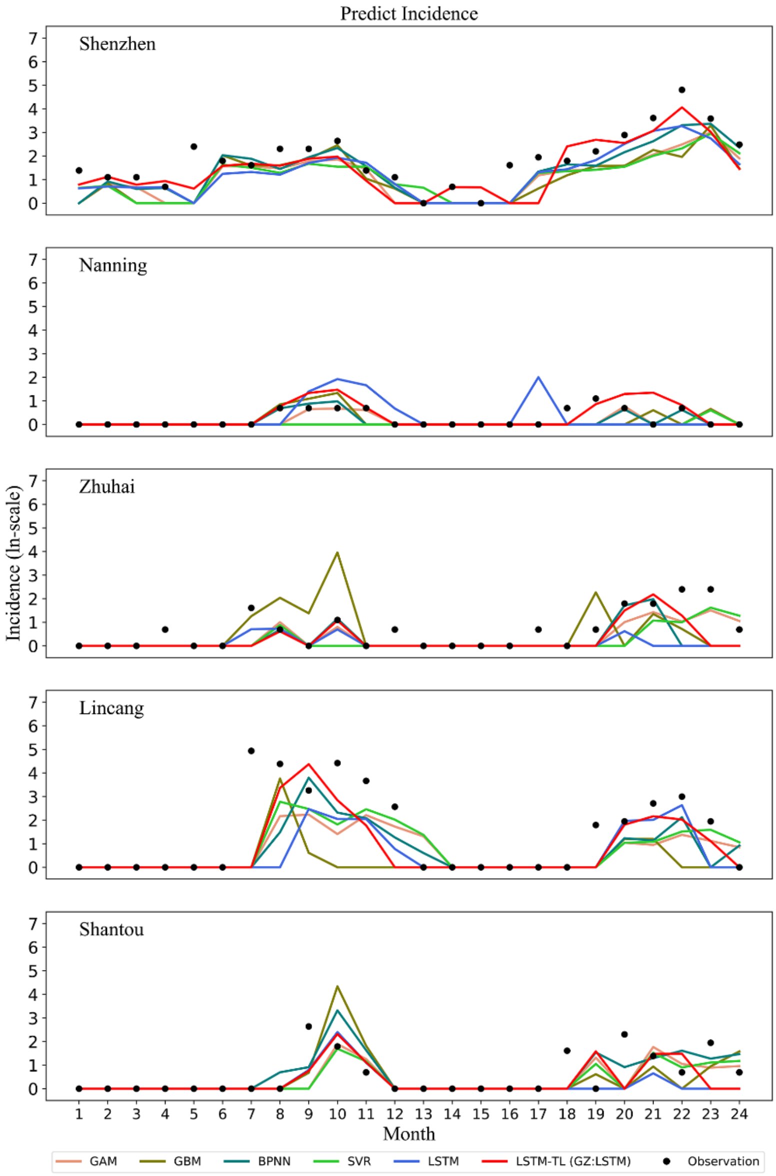

Figure S3. Prediction dengue cases in the last 24 months by the LSTM model, BPNN model, GBM model, GAM model, SVR model. Comparison of 24-month predictions for 2017 to 2018 in Shenzhen, Nanning, Zhuhai, Lincang, and Shantou.

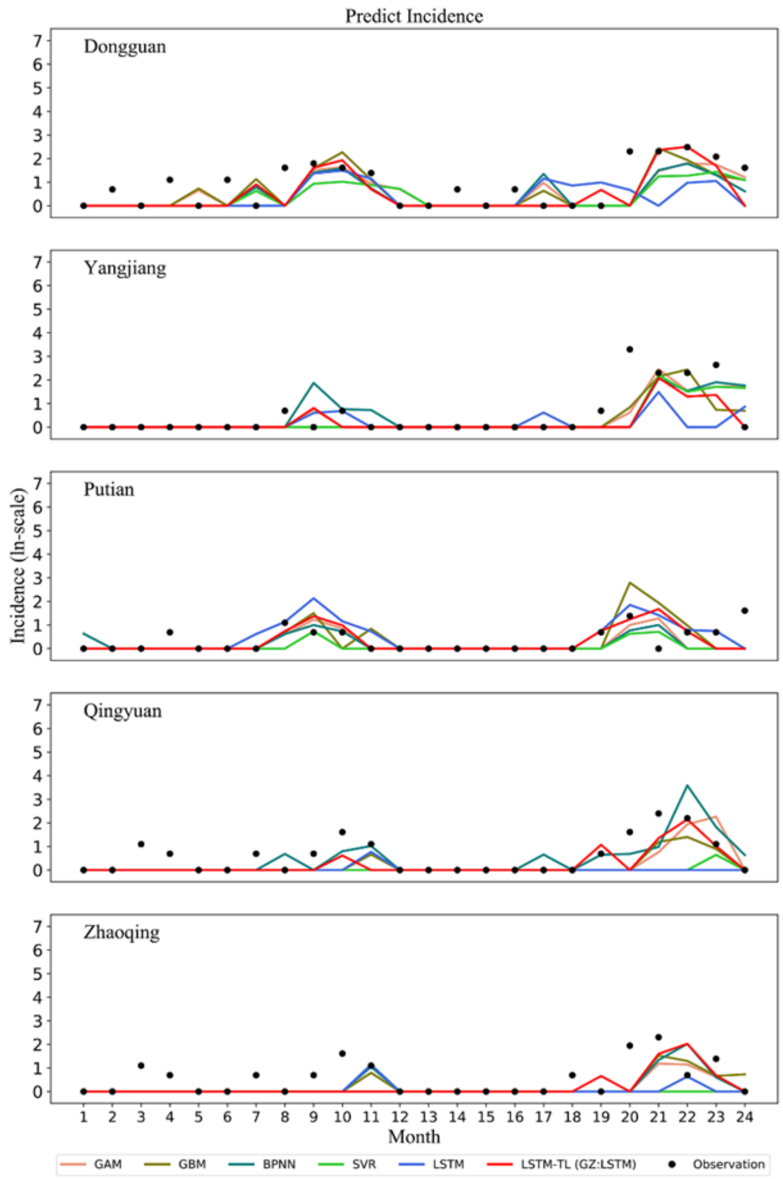

Figure 4. Prediction dengue cases in the last 24 months by the LSTM model, BPNN model, GBM model, GAM model, SVR model. Comparison of 24-month predictions for 2017 to 2018 in Dongguan, Yangjiang, Putian, Qingyuan, and Zhaoqing.
